# Differential role of CCR2 in the dynamics of microglia and perivascular macrophages during prion disease

**DOI:** 10.1002/glia.22660

**Published:** 2014-03-19

**Authors:** Diego Gómez-Nicola, Sjoerd TT Schetters, V Hugh Perry

**Affiliations:** 1 Centre for Biological Sciences, University of SouthamptonUnited Kingdom

**Keywords:** ME7 model, microglial proliferation, CCR2^−/−^, circulating monocytes, neurodegeneration

## Abstract

The expansion of the microglial population is one of the hallmarks of numerous brain disorders. The addition of circulating progenitors to the pool of brain macrophages can contribute to the progression of brain disease and needs to be precisely defined to better understand the evolution of the glial and inflammatory reactions in the brain. We have analyzed the degree of infiltration/recruitment of circulating monocytes to the microglial pool, in a prion disease model of chronic neurodegeneration. Our results indicate a minimal/absent level of CCR2-dependent recruitment of circulating monocytes, local proliferation of microglia is the main driving force maintaining the amplification of the population. A deficiency in CCR2, and thus the absence of recruitment of circulating monocytes, does not impact microglial dynamics, the inflammatory profile or the temporal behavioral course of prion disease. However, the lack of CCR2 has unexpected effects including the failure to recruit perivascular macrophages in diseased but not healthy CNS and a small reduction in microglia proliferation. These data define the composition of the CNS-resident macrophage populations in prion disease and will help to understand the dynamics of the CNS innate immune response during chronic neurodegeneration.

## Introduction

The determination of the relative contribution of local proliferation of microglial cells vs. the infiltration of bone-marrow derived progenitors during CNS pathology is a matter of intense debate. The recruitment of circulating progenitors or monocytes has been evidenced in models of neurodegeneration (Prinz and Mildner, 2011) highlighting the different roles of these cells and resident microglia in, for example, regulating amyloid [(Aβ) clearance in model of Alzheimer's disease (AD)] (Simard et al., 2006). However, the current trend supports *in situ* microglial proliferation as the major mechanism regulating microglial turnover, with little or no contribution of circulating progenitors (Gomez-Nicola et al., 2013; Lawson et al., 1992; Mildner et al., 2011). Circulating Ly6C^hi^CCR2^+^ monocytes are able to infiltrate the brain and influence pathology under certain conditions (brain preconditioning; Mildner et al., [Bibr b28],[Bibr b27]). CCR2^−/−^ mice have a greatly decreased Ly6C^hi^CCR2^+^ monocyte population and recruitment to the CNS parenchyma is CCR2-dependent making them a valuable model in which to study the role of recruited monocytes in chronic neurodegeneration (Mildner et al., 2007). Previous studies using CCR2^−/−^ mice reported decreased survival and increased Aβ load in APP or APP/PS1 transgenic mice and, to some extent, a reduction of the microglial numbers in the brain (El Khoury et al., 2007; Naert and Rivest, 2011). More recently, the increase in Aβ deposition was correlated with a defect in perivascular macrophages, rather than an impact over the microglial population (Mildner et al., 2011). However, it is unclear how the population of CNS-resident macrophages may be modulated in the context of other models of neurodegeneration, such as prion disease, where a massive expansion of the microglial population accounts for a significant pathological component of the disease (Gomez-Nicola et al., 2013).

Experimental models of prion disease are tractable laboratory models in which to study protein misfolding, progressive neuronal degeneration, synaptopathy, and the characteristic glial inflammatory response common to several neurodegenerative diseases (Ransohoff and Perry, 2009). In prion disease, a 10-fold expansion of the microglial population is observed, associated with the local proliferation of resident microglia driven through the CSF1R pathway (Gomez-Nicola et al., 2013). Abrogating microglial proliferation by selective targeting of the CSF1R activation has a protective effect and delays the progression of the clinical symptoms of the disease (Gomez-Nicola et al., 2013). Although CCR2 deficiency has been shown not to affect the survival of prion diseased mice (Tamguney et al., 2008), some studies suggest a contribution from bone-marrow derived cells to the microglial population (Priller et al., 2006) although this latter study was confounded by the effects of brain irradiation. Therefore, the degree of infiltration of circulating progenitors, even if small, needs to be evaluated in order to better understand the composition of the macrophage populations of the brain in progressive neurodegeneration.

We have studied the potential contribution of circulating monocytes or progenitors to the pool of macrophages/microglia in prion disease, using tracing techniques and investigated the role of CCR2 in the cell populations' dynamics. We provide evidence for a minimal/absent recruitment of circulating progenitors to the brain parenchyma, and report on the effects of CCR2 over the control of microglial and perivascular macrophage proliferation during the neurodegenerative process.

## Materials and Methods

### Experimental Model of Prion Disease

Female C57BL/6J (Harlan, Bicester, UK), c-fms-EGFP (Sasmono et al., 2003; macgreen), and C57BL/6J-CCR2^−/−^ mice (Menzies et al., 2012) were bred and maintained in local facilities. Mice expressing EGFP under the promoter of c-fms (CSF1R) are characterized by the expression of green fluorescence in microglial cells. Mice were housed in groups of 4–10, under a 12-h light/12-h dark cycle at 21°C, with food and water *ad libitum*. Prion disease was induced under anesthesia with a ketamine/rompun mixture (85 and 13 mg/kg, respectively), and injection of 1 μL of either ME7-derived (ME7-animals; 10% w/v) or normal brain homogenate (NBH-animals), injected stereotaxically and bilaterally in the brain at the coordinates from bregma: anteroposterior, −2.0 mm; lateral, −1.7 mm; depth, 1.6 mm. When required, mice were given an intraperitoneal (i.p.) injection of BrdU (Sigma-Aldrich; 7.5 mg/mL, 0.1 mL/10 g weight in sterile saline), on the 2 days before the end of the experiment. All procedures were performed in accordance with UK Home Office licensing.

### Tracing of Circulating Cells with CFDA

A stock solution of CFDA (Vybrant® CFDA SE Cell Tracer Kit, Molecular Probes) was prepared by dissolving 0.5 mg CFDA in 90 μL of dimethyl sulfoxide. A labeling solution (2% CFDA in sterile saline) was then prepared and after thorough mixing, injected (10 µL/g body weight) over a 5-min period via the tail vein of restrained conscious NBH (control) and ME7 (prion) mice, at 12 weeks post-induction. Similar results were obtained by injecting 100 µL intrasplenically after abdominal surgery, as previously reported (Bechmann et al., 2005). The animals were sacrificed at 48 h after the injection, to examine cell migration to the brain parenchyma.

### Behavioral Tests

CCR2-deficient (CCR2^−/−^) or wild-type (WT) mice treated with NBH or ME7 (*n* = 6/group), were tested weekly on behavioral tasks from the 13th week post-injection, previously demonstrated to detect the onset of behavioral dysfunction (Guenther et al., 2001): open-field locomotor activity, burrowing activity, and motor performance on an inverted screen were assessed.

### Open-Field Locomotor Activity

The open-field tests were carried out using activity monitoring software (Med Associated). The mice were placed in individual cages of 27 × 27 × 0.3 cm for a period of 3 min, to further analyze the total distance travelled (cm) and the number of ambulatory counts, using the average speed as an internal control of the mouse motor abilities, during the test period (3 min).

### Burrowing

Plastic cylinders, 20 cm long and 6.8 cm in diameter were filled with 190 g of normal diet food pellets and placed in individual mouse cages. Mice were placed individually in the cages overnight, weighting the remaining pellets at the end of the session, and calculating the amount displaced (“burrowed”). The mice were then returned to their home cage.

### Inverted Screen

The inverted screen is a 43-cm^2^ of wire mesh consisting of 12 mm^2^ of 1 mm diameter wire, surrounded by a 4-cm wooden bead to prevent the mouse from escaping the screen. Each mouse was placed in the center of the square and the screen inverted over 2 s, with the head of the mouse declining first. The screen was held steadily 20–30 cm above a padded surface. The time at which the mouse fell off was noted, or the mouse was removed when reaching the maximum time of the assay (120 s; Guenther et al., 2001).

### Body Weight and Late-Stage Clinical Signs of Disease

Body weights of all mice were monitored on a weekly basis from 8 weeks post-injection. Terminal disease was defined as a humane end-point of a loss of ≥15% body weight and/or the development of severe clinical signs; at this point animals were sacrificed.

### Immunohistochemistry

Coronal hippocampal sections were cut from paraformaldehyde-fixed, frozen, or fresh brains. Mice perfusion, tissue processing and immunohistochemical analysis was performed as previously described (Gomez-Nicola et al., [Bibr b15],[Bibr b12]), using the following primary antibodies: goat anti-Iba1 (Wako), rat anti-CD11b (ABD Serotec), chicken anti-GFP (Abcam), mouse anti-GFAP (Millipore), rat anti-CD34 (Abcam), rat anti-CD34 (BD Biosciences), rat anti-Ly6c (ABD Serotec), rabbit anti-laminin (Sigma-Aldrich), mouse anti-BrdU (DSHB), rat anti-BrdU (Santa Cruz Biotechnologies), rabbit anti-PU.1 (Cell Signaling), rabbit anti-CCR2 (Abcam), rat anti-CD206 (Mannose receptor, AbD Serotec), and rat anti-MHCII (EBioscience). Following primary antibody incubation, the sections were washed and incubated with the appropriate biotinylated secondary antibody (Vector Labs), and/or with the appropriate Alexa 405, 488, or 568 conjugated secondary antibody or streptavidin (Molecular Probes). For light microscopy, the sections were visualized using diaminobenzidine (DAB) precipitation, in a Leica CTR 5000 microscope, coupled to a Leica DFC300FX microscope camera. When required, the DAB signal was enhanced with 0.05% nickel ammonium sulfate, producing a black precipitate. After immunofluorescence labeling, nuclei were visualized by DAPI staining and the sections were mounted with Mowiol/DABCO (Sigma-Aldrich) mixture. The sections were visualized on a Leica TCS-SP5 confocal system, coupled to a Leica CTR6500 microscope.

The general immunohistochemistry protocol was modified for the detection of BrdU, adding a DNA denaturation step with 2N HCl (30 min, 37°C), as previously described (Gomez-Nicola et al., [Bibr b14],[Bibr b12]).

### Quantification and Image Analysis

The quantification of antigen positive cells (i.e., Iba1+) or antigen positive area (i.e., GFAP) was performed after DAB immunohistochemistry (*n* = 4 fields/mouse, *n* = 4–6 mice/group), as previously described (Gomez-Nicola et al., [Bibr b13],[Bibr b12]). Data were represented as number of positive cells/mm^2^. All quantifications were performed with the help of the ImageJ image analysis software.

### Analysis of Gene Expression by RT-PCR

CCR2-deficient (CCR2^−/−^) or WT mice treated with NBH or ME7 (*n* = 4–6/group) were processed to obtain samples from the hippocampus or the thalamus by dissection under a microscope, after intracardiac perfusion with heparinized 0.9% saline. RNA was extracted using the RNAqueous®-Micro Kit (Life Technologies), quantified using Nanodrop (Thermo Scientific), to be retrotranscribed using the iScript cDNA Synthesis Kit (Bio-Rad), following manufacturer's instructions, after checking its integrity by electrophoresis in a 2% agarose gel. cDNA libraries were analyzed by qPCR using the iTaq Universal SYBR Green supermix (Bio-Rad) and the following custom designed gene-specific primers (Sigma-Aldrich): csf1 (NM_007778.4; FW, agtattgccaaggaggtgtcag, RV, atctggcatga agtctccattt), il34 (NM_001135100.1; FW, ctttgggaaacgagaatttggaga, RV, gcaatcctgtagttgatggggaag), csf1r (NM_001037859.2; FW, gcag taccaccatccacttgta, RV, gtgagacactgtccttcagtgc), pu.1 (NM_011355.1; FW, cagaagggcaaccgcaagaa, RV, gccgctgaactggtaggtga), c/ebpa (NM_007678.3; FW, agcttacaacaggccaggtttc, RV, cggctggcgacatacag tac), runx1 (NM_001111021; FW, caggcaggacgaatcacact, RV, ctcgtg ctggcatctctcat), irf8 (NM_008320; FW, cggggctgatctgggaaaat, RV, cacagcgtaacctcgtcttc), cd34 (NM_133654.3; FW, gccctacaggagaaaggct gggt, RV, gcccctcgggtcacattggc), c-kit (NM_021099.3; FW, cgtcttccg gcacaacggca, RV, tgagcagcggcgtgaacagag), sca1 (NM_009124.6; FW, cccggggtggccgtgatac, RV, agctggctggtccgctcagg), ccl2 (NM_011333.3; FW, ttaaaaacctggatcggaaccaa, RV, gcattagcttcagatttacgggt), ccr2 (NM_009915; FW, aggagccatacctgtaaatgcc, RV, tgtggtgaatccaatgccct), il1b (NM_008361.3; FW, gaaatgccaccttttgacagtg, RV, tggatgctctcatca ggacag), tgfb (NM_011577; FW, tgtacggcagtggctgaacc, RV, cgtttgg ggctgatcccgtt), mhc class II antigen A, alpha (H2-Aa) (mhcII; NM_010378.2; FW, agctctgattctgggggtcctcg, RV, ataaacgccgtctgtgact gact), and ym1 (NM_009892; FW, atggaagtttggacctgccc, RV, agtagc agccttggaatgtctt). Quality of the primers and the PCR reaction were evaluated by electrophoresis in a 1.5% agarose gel, checking the PCR product size. Data were analyzed using the 2-ΔΔCt method with Primer Opticon 3 software, using gapdh (NM_008084.2; FW, tgaacgggaagctcactgg, RV, tccaccaccctgttgctgta) as a housekeeping gene.

### Statistical Analysis

All experiments were designed and performed in accordance with the ARRIVE guidelines. Data were expressed as mean ± SEM and analyzed with the GraphPad Prism 5 software package (GraphPad Software). For all data sets, normality and homoscedasticity assumptions were reached, validating the application of the one-way or two-way ANOVA, followed by the Tukey *post hoc* test for multiple comparisons.

## Results

### Prion disease causes a mild expansion of the CD34^+^ population while driving no detectable recruitment of circulating progenitors to the brain parenchyma

**Figure 1:**
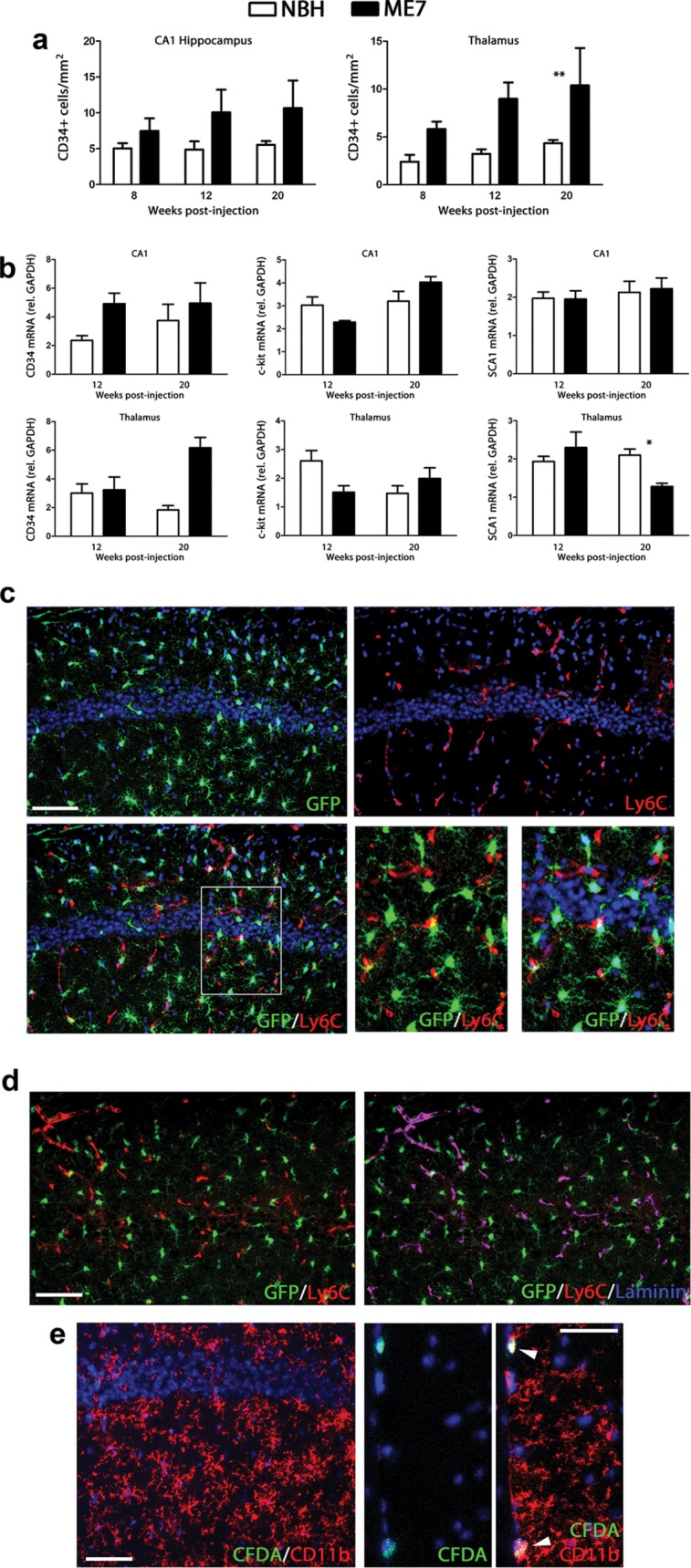
Analysis of the infiltration of peripheral monocyte precursors in prion diseased brain. (a) Immunohistochemical analysis of the expression of CD34 in the CA1 region of the hippocampus and the thalamus of prion disease (ME7) and control (NBH) mice. Quantified data expressed as mean ± SEM of the number of CD34^+^ cells/mm^2^. (b) Analysis of the expression of mRNA of CD34, c-kit and SCA1 in the hippocampus (CA1) and thalamus of prion disease (ME7) and control (NBH) mice. Expression of CD34, c-kit and SCA1 represented as mean ± SEM and indicated as relative expression levels using the 2−ΔΔCt method. (c) Analysis of the infiltration of Ly6c^+^ monocytes by double immunofluorescence for Ly6c (red) and GFP (microglia, green) in the CA1 region of the hippocampus of prion disease (ME7). (d) Analysis of the expression of Ly6c in blood vessels by triple immunofluorescence for Ly6c (red), GFP (microglia, green), and laminin (blue) in the hippocampus (CA1) of prion disease mice (ME7). (e) Analysis of the infiltration of peripheral cells (CFDA^+^, green) in the CA1 hippocampal layer (left) or the meningeal space (mid and right panels) in prion disease (microglia shown as CD11b^+^, red). White arrowheads indicate CFDA^+^/CD11b^+^ cells. Statistical differences: *P < 0.05, ** *P* < 0.01. Data were analyzed with a two-way ANOVA and a *post hoc* Tukey test. (c) Nuclei are stained with Hoechst (blue). (c, d) Fluorescent sections evaluated with confocal microscopy. Scale bar in (c, d) 50 µm; in (e) 20 µm.

Following our recent findings defining the control and consequences of microglial proliferation in prion disease (Gomez-Nicola et al., 2013), we now set out to characterize the potential contribution of infiltrated peripheral precursor cells to the disease. We analyzed the expression or localization of different markers of peripheral precursor cells or hematopoietic stem cells during the time course of prion disease (Fig. 1). First, we analyzed the expression of CD34^+^ during prion disease (Fig. 1A), an antigen reported as characteristic of proliferating microglial cells (Ladeby et al., 2005b). CD34^+^ cells show a trend to be more numerous in prion disease mice when compared with the NBH group, more evident in the thalamus at late-stage disease (Fig. 1A). A similar trend was observed in the other areas analyzed (data not shown), as well as at the mRNA level (Fig. 1B). The analysis of the mRNA expression of c-kit and sca1, markers for bone-marrow progenitors, showed no significant increase in prion diseased brain, when compared with the NBH controls (Fig. 1B).

Next, we analyzed the identity of Ly6c-expressing cells, an antigen characteristic of endothelial cells and infiltrated monocytes (Mildner et al., 2007; Prinz and Mildner, 2011). The expression of Ly6c (Fig. 1C; red) in prion diseased brains is absent from microglial cells (Fig. 1C; GFP^+^), as evidenced by confocal microscopy. Ly6c (Fig. 1D; red) is expressed in blood vessels, as evidenced by its colocalisation with laminin^+^ capillaries (Fig. 1D; blue) but not with GFP^+^ microglial cells (green). Using an alternative approach, labeling peripheral monocytes with the fluorescent tracer CFDA, we did not observe CD11b^+^ microglial cells (red) being generated from circulating precursors (CFDA^+^, green) in the parenchyma of the brain (Fig. 1E). CFDA^+^ cells expressing CD11b were found in the meninges (Fig. 1E, mid and right panels; white arrowheads) and in the perivascular space, at a similar frequency in both NBH and ME7 mice.

Our results show some expansion of the CD34^+^ cell population, independent of the recruitment of circulating progenitors to the microglial pool, supporting previous studies highlighting the important role of proliferation of resident microglial cells in prion disease (Gomez-Nicola et al., 2013).

### The expansion of the microglial population in prion disease is independent of the contribution of circulating monocytes

**Figure 2:**
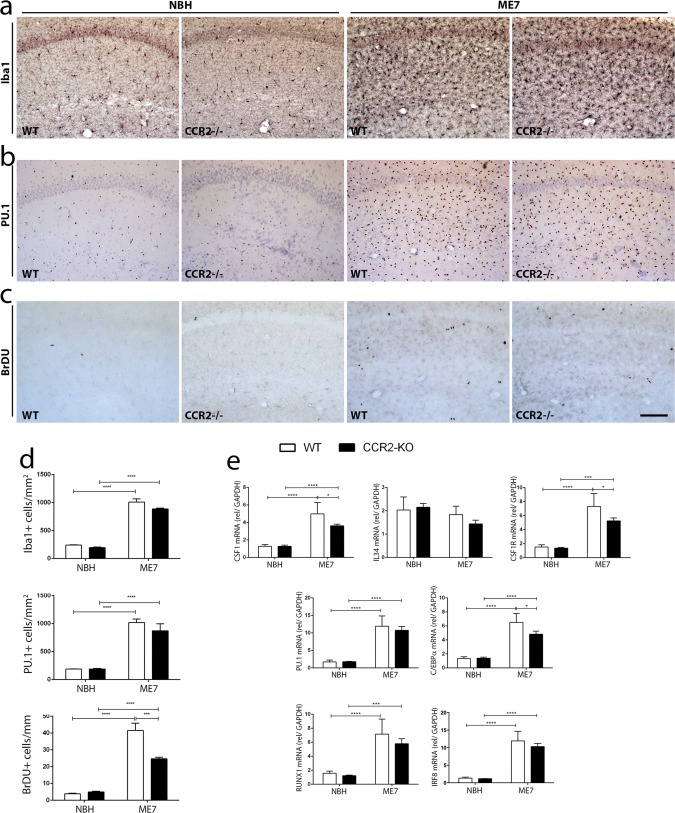
Characterization of the impact of CCR2 deficiency on the dynamics and regulation of microglial proliferation in prion disease. (a–c) Immunohistochemical analysis of the expression of Iba1 (a), PU.1 (b) and the incorporation of BrdU (c) in the CA1 region of the hippocampus of prion disease (ME7) and control (NBH) mice, at 20 weeks postinduction. (d) Quantification of the number of microglial cells (Iba1^+^, PU.1^+^) or proliferating cells (BrDU^+^); data expressed as mean ± SEM of the number of antigen+ cells/mm^2^. (e) Analysis of the expression of mRNA by qPCR of CSF1, IL34, CSF1R, PU.1, C/EBPα, RUNX1, and IRF8 in the hippocampus of prion disease (ME7) and control (NBH) mice, at 20 weeks postinduction. Expression of mRNA represented as mean ± SD and indicated as relative expression levels to GAPDH using the 2−ΔΔCt method. Statistical differences: *P < 0.05, ***P < 0.001, and ****P < 0.0001. Data were analyzed with a two-way ANOVA and a *post hoc* Tukey test. (a, b) Nuclei are stained with Neutral Red. Scale bar in (a–c) 50 µm (in c). [Color figure can be viewed in the online issue, which is available at wileyonlinelibrary.com.]

Given the recent evidence that the Ly-6C^hi^CCR2^+^ population of circulating monocytes infiltrates the brain during pathology in a CCR2-dependent manner (Prinz and Mildner, 2011), we analyzed the progression of prion disease in CCR2^−/−^ mice. The microglial population (Iba-1^+^ cells) expanded to similar levels in the hippocampus (HC) of WT and CCR2^−/−^ mice, in response to prion disease (ME7), when compared with NBH controls (Fig. 2A,D). The transcription factor underlying microglial proliferation in prion disease, PU.1 (Gomez-Nicola et al., 2013), is similarly expressed in both WT and CCR2^−/−^ mice, and is upregulated in response to prion disease (Fig. 2B,D). Cell proliferation (BrdU^+^ cells) increases upon injection of ME7, but the proliferative fraction of the expanding microglia population (Brdu^+^/Iba-1^+^ cells) shows a 2.64-fold increase in WT ME7-animals, while CCR2^−/−^ ME7-animals show a significantly smaller 1.11-fold increase (Fig. 2C,D). The analysis of mRNA expression by qPCR correlated with the results obtained by immunohistochemistry (Fig. 2E). The mRNA expression of PU.1 and C/EBPα, transcription factors regulating microglial proliferation, increases to similar levels in the HC of both WT and CCR2^−/−^ mice with prion disease (ME7, 20wpi), although C/EBPα mRNA expression was found to be relatively less expressed in CCR2^−/−^ ME7-animals, when compared with the WT ME7-animals (Fig. 2E) consistent with the smaller increase in Brdu labeled cells in the CCR2^−/−^ ME7-animals. The transcription factors RUNX1 and IRF8, involved in the proliferation and lineage commitment of microglia (Ginhoux et al., 2010; Kierdorf et al., 2013), were found to be upregulated in animals with prion disease, with no differential effect of the CCR2^−/−^ background (Fig. 2E). The expression of CSF1 and its receptor CSF1R show similar patterns, appearing upregulated in prion disease, with the CCR2^−/−^ background having a less pronounced effect (Fig. 2E). The expression of IL34, an alternative ligand for CSF1R, shows no significant change with the different genetic backgrounds (WT vs. CCR2^−/−^) or experimental groups (NBH vs. ME7) used at the 20W time-point, in accord with previously reported results (Gomez-Nicola et al., 2013).

These results support the notion that the expansion of the microglial population in prion disease is independent of the recruitment of circulating monocytes, and is largely if not exclusively due to proliferation of resident microglial cells. We observed, however, that lack of CCR2 had a small but significant effect on the microglia proliferative response.

### CCR2 Deficiency Inhibits the Expansion of the Population of Perivascular Macrophages in Prion Disease

**Figure 3:**
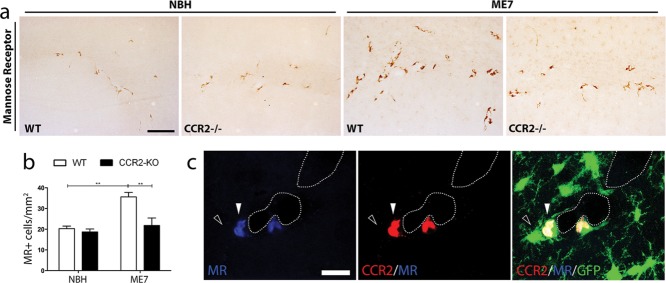
Characterization of the impact of CCR2 deficiency on the perivascular macrophage population. (a) Immunohistochemical analysis of the expression of Mannose receptor (MR; CD206) in the CA1 region of the hippocampus of prion disease (ME7) and control (NBH) mice, at 20 weeks postinduction. (b) Quantification of the number of MR^+^ cells; data expressed as mean ± SEM of the number of antigen^+^ cells/mm^2^. (c) Confocal analysis of the expression of CCR2 (red) by triple immunofluorescence for MR (blue) and GFP (microglia, green) in the hippocampus [CA1 of prion disease mice (ME7)]. Statistical differences: ** *P* < 0.01. Data were analyzed with a two-way ANOVA and a *post hoc* Tukey test. Scale bar in (a) 50 µm; in (c) 10 µm.

Following recent reports highlighting the role of perivascular macrophages during chronic neurodegeneration (Mildner et al., 2011), we analyzed this population in the prion diseased animals (Fig. 3). Prion disease causes an expansion of the perivascular macrophage population [Mannose receptor (MR)^+^; Fig. 3A,B], when compared with NBH mice. Although CCR2 deficiency does not impact the renewal of the perivascular macrophage population in naïve or control conditions, it inhibits the expansion of the population as observed in comparing the WT and CCR2^−/−^ ME7-animals (Fig. 3A,B). We did not find a significant proliferative fraction of perivascular macrophages in response to prion disease (data not shown), validating the hypothesis that this population is renewed/expanded from circulating progenitors (Mildner et al., 2011). To better understand the differential responses of the microglial and perivascular macrophages populations (Figs. 2 and 3) we investigated the expression of CCR2 in brains of WT ME7-animals (Fig. 3C). Confocal microscopy analysis shows CCR2 expression (red) in perivascular macrophages (MR^+^, blue), but not in parenchymal microglial cells (GFP^+^) in WT ME7-animals (Fig. 3C) and was absent in CCR2^−/−^ mice (data not shown).

These results support a CCR2 dependent expansion of the perivascular macrophage population in prion disease, but not in the healthy brain.

### CCR2 Deficiency Does Not Modify the Microglial Inflammatory Response to Prion Disease

**Figure 4:**
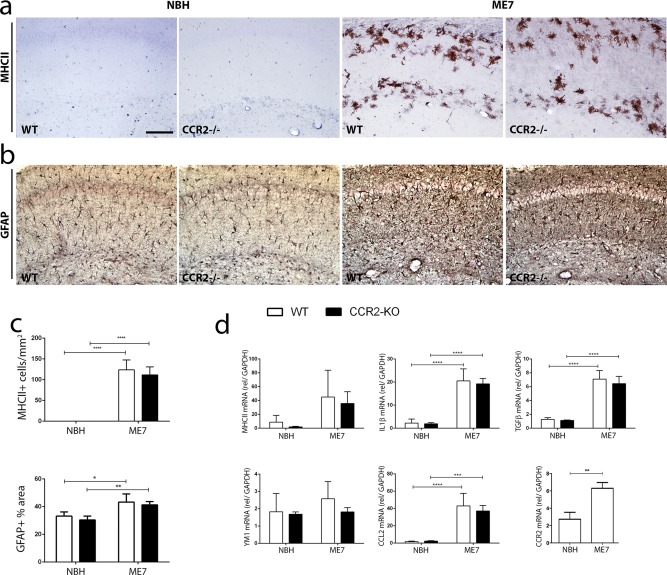
Characterization of the impact of CCR2 deficiency on the inflammatory and gliotic response in prion disease. (a, b) Immunohistochemical analysis of the expression of MHCII (a) and GFAP (b) in the CA1 region of the hippocampus of prion disease (ME7) and control (NBH) mice, at 20 weeks post-induction. (c) Quantification of the number of MHCII^+^ or GFAP^+^ cells; data expressed as mean ± SEM of the number of antigen+ cells/mm^2^. (d) Analysis of the expression of mRNA by qPCR of MHCII, IL1β, TGFβ, YM1, CCL2, and CCR2 in the hippocampus of prion disease (ME7) and control (NBH) mice, at 20 weeks postinduction. Expression of mRNA represented as mean ± SD and indicated as relative expression levels to GAPDH using the 2−ΔΔCt method. Statistical differences: * *P* < 0.05, ** *P* < 0.01, *** *P* < 0.001, and **** *P* < 0.0001. Data were analyzed with a two-way ANOVA and a *post hoc* Tukey test. Scale bar in (a, b) 50 µm in (a). [Color figure can be viewed in the online issue, which is available at wileyonlinelibrary.com.]

We next aimed to address the impact of CCR2 deficiency on the generation of the microglial inflammatory response in prion disease (Fig. 4). We first analyzed the expression of MHCII by immunohistochemistry, and found increased expression in ME7-animals both with a WT or CCR2^−/−^ background (Fig. 4A,C), in accord with the results obtained at the mRNA level (Fig. 4D). Quantitative PCR analysis of inflammatory mediators shows a significant increase of the expression of the proinflammatory cytokine IL1β in ME7-animals when compared to NBH-animals (Fig. 4D), regardless of the genetic background (WT vs. CCR2^−/−^). Similar results were obtained for the expression of the anti-inflammatory cytokine TGFβ, found to be upregulated in response to prion disease, independent of the deficiency of CCR2 (Fig. 4D). The expression of the marker of M2-like inflammatory activation, YM1, was in accord with previously reported data (Gomez-Nicola et al., 2013), showing non-significant change with prion disease, and these results were not differentially affected by the WT or CCR2^−/−^ background (Fig. 4D). Both the chemokine CCL2 and its receptor CCR2 were found to be upregulated in WT ME7-animals (Fig. 4D), while CCR2 deficiency did not significant alter the expression of CCL2.

Given the results regarding microglial activation in response to prion disease upon CCR2 deficiency, we wanted to analyze its impact on the astroglial response. Using immunohistochemical analysis, we found an increased expression of GFAP in ME7-animals, with no observed differences when comparing WT vs. CCR2^−/−^ mice (Fig. 4B,C).

### CCR2 Deficiency Does Not Modify the Pathological Course of Prion Disease

**Figure 5:**
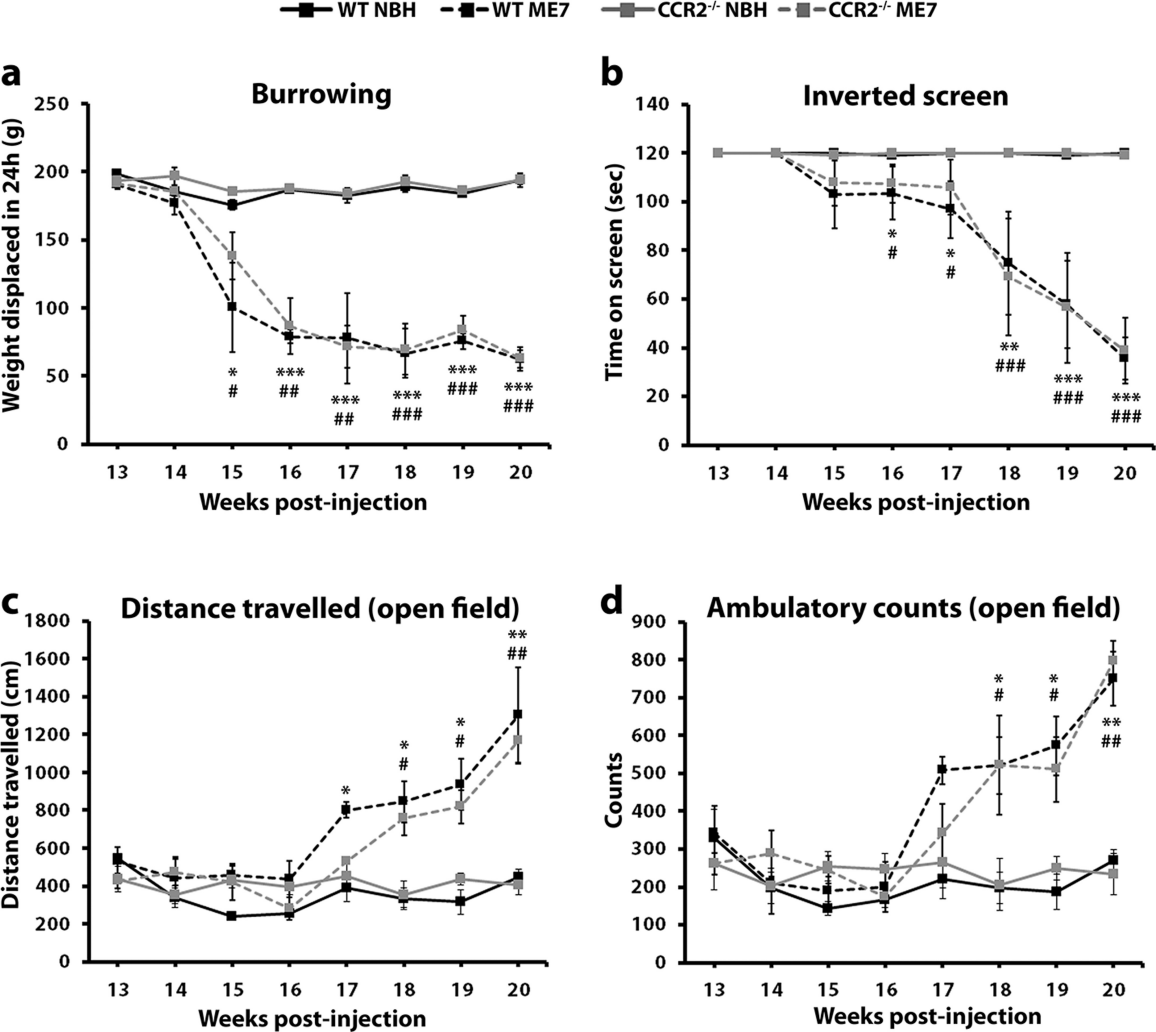
Effect of CCR2 deficiency over the behavioral progression of prion disease. Time-course of the behavioral responses observed in control (NBH) or prion diseased mice (ME7), with a WT or CCR2^−/−^ genetic background. (a) Effect of the different experimental groups on the burrowing behavior, measured as weight displaced (g) off the tube in 24 h. (b) Effect of the different experimental groups on the motor performance and coordination, measured as time spent (seconds) on the inverted screen test. (c, d) Effect of the different experimental groups on the locomotor activity, measured as distance travelled (cm) and ambulatory counts in the open field test. Statistical differences: WT NBH vs. WT ME7 *P < 0.05, ** *P* < 0.01, *** *P* < 0.001; CCR2^−/−^ NBH vs. CCR2^−/−^ ME7 ^#^ *P* < 0.05, ^##^ *P* < 0.01, ^###^ *P* < 0.001. Data were analyzed with a two-way ANOVA and a *post hoc* Tukey test.

Following the observations that circulating progenitors do not contribute to the microglial pool (Figs. 1 and 2) and that CCR2 deficiency does not modify the inflammatory reaction in prion disease except in the perivascular compartment (Fig. 4), we examined the pathological course of the ME7 model in WT vs. CCR2^−/−^ mice using behavioral techniques (Fig. 5), to address the overall contribution of CCR2 to the disease. CCR2 deficiency did not affect the normal decay in burrowing activity observed from the 14th week in WT ME7-animals, maintaining a similar behavioral trend (Fig. 5A). Similarly, we did not observe any genotype-dependent difference in the effect of prion disease on the loss of motor strength and motor coordination evidenced by the inverted screen task, when compared with NBH controls (Fig. 5B). The behavioral course of prion disease is also characterized by a phase of hyperactivity, observed at similar levels in WT or CCR2^−/−^ ME7-animals and identified by an increase in the distance travelled and the ambulatory counts in the open field task (Fig. 5C,D). We did not observe any difference in the behavior of control mice (NBH) from a WT or CCR2^−/−^ background in the reported tests.

These results indicate that CCR2 deficiency does not impact the pathological course of prion disease, thus supporting the hypothesis that there is little or no contribution of circulating progenitors to the microglial pool and indicating a minimal role of the CCL2-CCR2 system on this disease.

## Discussion

The expansion of the microglial population is a characteristic of the progression of most brain pathologies. We provide evidence for a minimal/absent recruitment of circulating progenitors or monocytes during prion disease: recruited monocytes make a negligible, if any, contribution to the expansion of the microglial population, the inflammatory reaction and the behavioral progression of the neuropathology. We recently showed that the proliferation of microglial cells during prion disease is controlled by the activity of the CSF1R pathway, and blocking this receptor has a beneficial effect by delaying the progression of the pathology (Gomez-Nicola et al., 2013). Our results support the hypothesis of local proliferation being responsible for the expansion of the microglial population (Ajami et al., 2007; Mildner et al., [Bibr b28],[Bibr b27]), contradicting the earlier observed recruitment of peripheral bone-marrow progenitors (Priller et al., 2006; Williams et al., 1995). The recruitment of circulating progenitors to sites of evolving chronic neurodegeneration is rare, and only seen where an experimental alteration of the system, for example where irradiation, compounds the cell migration (Mildner et al., 2011; Prinz and Mildner, 2011). Our results may indicate the existence of a minor CD34^+^ microglial population during neurodegeneration, previously identified in models of acute neural injury (Ladeby et al., 2005a). Expression of the stem cell antigen CD34 has been linked to a marked ability of self-renewal and as a characteristic of proliferating resident microglia, and not necessarily of infiltrating bone-marrow precursors (Ladeby et al., 2005b). However, further experiments would be necessary to support the existence of a highly proliferative CD34^+^ microglial subpopulation in prion disease.

The evidence reported here shows that there is minimal impact of CCR2 deficiency on prion disease. These results are in line with previously reported data indicating that CCR2 deficiency does not modify prion incubation times or survival (Tamguney et al., 2008). We observe a similar expansion of the microglial pool (Iba1^+^ or PU.1^+^ cells) in response to prion disease in mice with a WT or CCR2^−/−^ background. We did however observe an impact of CCR2 deficiency on the expansion of the perivascular macrophage population driven by prion disease (Galea et al., 2005), which is somewhat different compared to the results reported for the APP/PS1 model of AD (Mildner et al., 2011), even though the innate immune response in APP transgenics and experimental models of prion disease share many similarities. Different detection methods could be one reason for this difference and an in-depth analysis of the expression of specific markers of perivascular macrophages, like CD163 or mannose receptor (CD206; Galea et al., [Bibr b9],[Bibr b8]; Hawkes and McLaurin, 2009) will help to understand the dynamics of this population in APP models. However, the behavioral results obtained in this work indicate a negligible role of the perivascular macrophage population during the progression of prion disease. This is indicated by the fact that abolishing the expansion of the perivascular macrophage population in response to prion disease in CCR2 deficient mice had no impact over the progression of the pathology.

The chemokine receptor CCR2 can bind all the monocyte chemoattractant proteins, although it is the only established high-affinity receptor for CCL2 (Charo et al., 1994; Huang et al., 2005). Both CCL2 and CCR2 are upregulated in prion disease, as reported in the present work and previously (Felton et al., 2005), indicating a potential role for of this receptor-ligand interaction. CCL2^−/−^ mice show an amelioration of the behavioral deficits and a mild increase of survival when infected with the ME7 prion strain, without affecting the microglial population (Felton et al., 2005). These effects were not replicated by genetic deletion of CCR2. Instead we observed a small reduction of the expression of the mitogenic pathways controlling microglial proliferation (CSF1R). The activity of the CCL2-CCR2 pathway has been previously shown to stimulate microglial proliferation (Hinojosa et al., 2011), although this reported link of CCR2 with microglial proliferation was not observed in CSF1-treated cells isolated from CCR2^−/−^ mice (Mildner et al., 2007). Although we observed a reduction in microglial proliferation it did not impact the endpoint expansion of the cell population, indicating that other balancing forces could be modulated by CCR2 deficiency, as for example apoptosis of microglial cells (Gomez-Nicola et al., 2013). Our results indicating a minimal impact of CCR2 deletion on the inflammatory state of microglial cells are in accord with previously reported evidence with microglia isolated from CCR2^−/−^ mice, which show no differences from WT microglia with regard to expression of IL6, TNFα, or MCH class II after stimulation with LPS or Pam_3_CSK_4_ (Mildner et al., 2007). It is also interesting to be noted that CCR2 was not detected on microglial cells throughout development (Mizutani et al., 2012). Therefore, the effects of the activation of the CCL2/CCR2 system on the control of cell proliferation would need to be further explored, to better understand the discrepancies present in the literature.

CCL2 is expressed by astrocytes through the TNF-α/JNK pathway (Gao et al., 2009), activated microglia in senile Aβ plaques, and reported to be expressed by some neurons (Hickman and El Khoury, 2013). Although the expression of CCR2 in the brain has been reported in neurons in the spinal cord (Gao et al., 2009), in certain regions of the brain (Bose and Cho, 2013), including the neurogenic niches (Tran et al., 2007), its expression in the brain has been defined by other authors to be specific to infiltrating precursors, and is not present on any other cell from the microglial or neuroepithelial lineage (Mizutani et al., 2012; Saederup et al., 2010). We here report CCR2 expression in perivascular macrophages, and we hypothesize that a deficient or aberrant signal derived from the perivascular compartment might be responsible for the observed decrease in the proliferative activity of microglia in CCR2^−/−^ ME7 mice. The impact of CCR2, independent of its effect on the microglia, has been previously described in the brain. It has been reported that CCL2, in a CCR2-dependent manner, can be neuroprotective against HIV-1 transactivator protein (Tat) toxicity in rat primary midbrain neurons and after intrastriatal injection of Tat (Yao et al., 2009). Similarly, CCR2 deficiency significantly reduces the generation of neuropathic pain (Gao et al., 2009). In the MPTP model of murine Parkinson's Disease (PD), CCL2 is upregulated in astrocytes and neurons, but the absence of CCL2 and CCR2 does not protect against loss of dopaminergic neurons in the striatum (Kalkonde et al., 2007). More recently, CCR2 deficiency was shown to prevent hippocampus-dependent spatial learning and memory impairments induced by cranial irradiation, highlighting the potential neuron-specific functions of this receptor (Belarbi et al., 2013; Raber et al., 2013). These contrasting views might be explained by alternative ligands, besides CCL2: binding and signaling through CCR2 can be achieved by CCL2, CCL7, CCL8, CCL12, and CCL13 (Bose and Cho, 2013). For example, CCL8 was reported to elicit the CCR2-mediated neuroprotective effects after irradiation (Belarbi et al., 2013). Together with the findings reported here, the literature supports a re-evaluation of the expression levels and activity of the system of CCR2 and its ligands in the brain, to better understand its functions in different brain cells.

To summarize, our results support the evidence that the expansion and activation of the microglial population in chronic neurodegeneration is independent of the recruitment of circulating monocytes. We highlight the relevance of CCR2 for the control of the dynamics of the perivascular macrophage population. Our results clearly define the existence of three compartments, the blood, the perivascular compartment and the brain parenchyma that regulate immune-to-brain communication. Careful evaluation of the dynamics between these compartments will be key to understanding the influence of the innate immune response on the progression of chronic neurodegeneration.
